# ‘It was like coming back from the clouds’: a qualitative analysis of the lived experience of overdose consequent to drug use among a cohort of people who use drugs in Scotland

**DOI:** 10.1186/s12954-024-01033-7

**Published:** 2024-06-07

**Authors:** Christopher J. Byrne, Fabio Sani, Teresa Flynn, Amy Malaguti

**Affiliations:** 1 Division of Molecular and Clinical Medicine, School of Medicine, Ninewells Hospital and Medical School, University of Dundee, Dundee, UK; 2 Directorate of Public Health, NHS Tayside, Kings Cross Hospital, Dundee, UK; 3 Division of Psychology, School of Humanities, Social Sciences and Law, University of Dundee, Scrymgeour Building, Dundee, UK; 4 Hillcrest Futures, Explorer Road, Dundee, UK; 5 Tayside Drug and Alcohol Recovery Psychology Service, NHS Tayside, Dundee, UK

**Keywords:** Overdose, Qualitative, Lived experience, People who use drugs.

## Abstract

**Background:**

Globally, non-fatal overdose (NFOD) rates consequent to drug use, typically opioids, continue increasing at a startling rate. Existing quantitative research has revealed myriad factors and characteristics linked to experiencing NFOD, but it is critically important to explore the lived context underlying these associations. In this qualitative study, we sought to understand the experiences of NFOD among people who use drugs in a Scottish region in order to: enhance public policy responses; inform potential intervention development to mitigate risk; and contribute to the literature documenting the lived experience of NFOD.

**Methods:**

From June to July 2021, two peer researchers conducted face-to-face semi-structured interviews with people who use drugs who had experienced recent NFOD attending harm reduction services in Tayside, Scotland. These were transcribed verbatim and evaluated using thematic analysis with an inductive approach which had an experiential and essentialist orientation.

**Results:**

Twenty people were interviewed across two sites. Of those, 15 (75%) were male and mean age was 38.2 (7.7) years. All had experienced at least one NFOD in the prior six months, and all reported polydrug use. Five themes were identified, within which 12 subthemes were situated. The themes were: social context; personal risk-taking triggers; planned and impulsive consumption; risk perception; and overdose reversal. The results spoke to the environmental, behavioural, cognitive, economic, and marketplace, factors which influence the context of NFOD in the region.

**Conclusions:**

A complex interplay of behavioural, psychological, and situational factors were found to impact the likelihood of experiencing NFOD. Structural inequities which policy professionals and civic leaders should seek to remedy were identified, while service providers may seek to reconfigure healthcare provision for people who use drugs to account for the interpersonal, psychological, and social factors identified, which appear to precipitate NFOD.

**Trial registration:**

Not applicable.

**Supplementary Information:**

The online version contains supplementary material available at 10.1186/s12954-024-01033-7.

## Vernacular notes

We include the following to facilitate understanding:

- Aye: yes.
- Didnae: did not.
- Ken / ken what I mean: know/know what I mean.
- Kick about with: associate/socialise with.
- Kit: drugs.
- Pure: really.
- Rattle: withdrawal.
- Units: quantity of a given drug (e.g. heroin).
- Vallies: ‘street’ benzodiazepines.
- Wee: small/little.

## Introduction

Globally, non-fatal overdose (NFOD) rates consequent to drug use, typically illicit opioids, are increasing at a startling rate: an estimated 3.2 (1.8–5.2) million people who use drugs experienced at least one NFOD in the last year, with up to 42% (35-48%) ever experiencing NFOD [1, 2]. NFOD has been shown to precipitate future fatal overdose in a dose-response type relationship [3]. The frequency of NFOD among people who use drugs (PWUD) – and high potential for subsequent fatality – is compounded upon additional health risks which manifest in higher rates of, for example, skin and soft tissue infections, and conditions such as viral hepatitis, botulism, clinical depression, and post-traumatic stress disorder [4–6]. Given the grave ongoing risks to the health of PWUD that survive overdose, and the complex bereavement experiences of those whose loved ones do not survive, connected with the stigma of the context of the death, it is vital to understand how NFOD is experienced, the contextual and environmental factors which drive it, and how these factors may be modified to prevent it [7].

The rise in overdose fatalities in recent decades has been associated with a triple-wave epidemic characterised by widespread opioid use; first of prescription opioid pills, then heroin use, and subsequently synthetic opioids including fentanyl, and fentanyl analogues, often combined with or switched for heroin [8]. This focus of this epidemic in North America has led to nearly 600,000 fatalities in the last twenty years, with this predicted to rise to 1.2 million by 2030, whilst increasingly fatal overdoses in the UK track along similar lines [9]. Overdose and subsequent fatalities in Scotland, where this study was conducted, and some other UK nations, are driven by widespread market availability of heroin and other opioids in the street drug scene, alongside easily accessible psychoactive drugs like benzodiazepines of variable quality (e.g. diverted vs. ‘street benzos’), and newer synthetic opioids called nitazenes, typically mis-sold as oxycodone [10–13]. Fentanyl has also been detected in the drug supply – though less common than in North America – whilst stimulant use (e.g. crack cocaine) is common [14]. Existing quantitative research has revealed myriad factors and characteristics linked to experiencing NFOD. For example: poly-drug use; recently ceased addictions treatment; involuntary drug treatment; experience of sex work; opioid agonist therapy (OAT) discontinuity and re-entry; and injecting in public spaces, have all been associated with higher likelihood of NFOD in British, North American, and European contexts [15–20]. Over and above identifying these factors, it is critically important to explore the ‘why’ and ‘how’ underlying these associations, by considering people’s lived experience. This can enhance our understanding of risk knowledge, interpretation of events, intentionality, and other contextual factors which may drive NFOD risk, alongside the many known psychosocial risk factors [21]. A 2015 study examined NFOD experience among non-medical prescription opioid users, and found participants were unaware of strategies for avoidance and response, and had little awareness of wider risks associated with it [22]. Other research involving recreational opioid users in England, half of whom had experienced NFOD, highlighted that just 32% of participants recognised established risk factors for NFOD at baseline [23]. Alluding to the psychological distress often experienced by PWUD, research which examined NFOD among people recently liberated from prison reported participants intentionally overdosing as a coping mechanism for stress and anxiety [24]. Whilst young adults and adolescents have expressed difficulty remembering whether they have experienced NFOD, with some intentionally seeking a ‘borderline overdose’ to achieve maximum high without dying [25]. Encompassing many of these issues, a North American study found social dynamics; uncertainty around drug supply; balancing polydrug use; and emotional pain, to be critical overarching factors reported as impacting upon NFOD experience [26].

In Scotland, few studies have explored such contextual issues with PWUD, despite fatal overdose rates being among the highest per capita in Europe, and higher than many international settings despite variable recent trends [27, 28]. Although drug-checking services may help in reducing harms associated with unregulated drug supply, in Scotland none are currently available despite being setup in other UK nations (although ongoing work is exploring this) [29, 30]. Therefore, OAT has been a critical first-line harm reduction measure, and has been shown to protect against overdose fatality in Scotland, but not enough to meaningfully arrest temporal increases observed since 2011 [31]. Recent work has shown that in Tayside, the Scottish region where this work was based, overdose fatalities are also high, while recent NFOD was disclosed by 36% of PWUD locally in a recent study [32]. Furthermore, of the 78 people who fatally overdosed in the region in 2021, 59% were known to have had at least one prior NFOD, with 13% experiencing one in the month prior to their death [33]. Overdose and associated mortality is of such critical public health concern that in 2022 the Scottish Government created a new ministerial post to oversee a national ‘mission’ to reduce deaths consequent to illicit drug use, which included obligatory implementation of service reform across the National Health Service (NHS) [34, 35]. It is in this context that we sought to understand the lived experiences of NFOD among PWUD in Tayside, to enhance current public policy responses, inform potential intervention development to mitigate risk, and contribute to the literature documenting the lived experience of NFOD.

## Methods

### Setting

This study was conducted in Tayside, Scotland. Tayside is a geographic region in the East of Scotland, home to approximately 418,000 people. Healthcare is provided free at the point of need by the NHS. The NHS, with partners from the charity sector, operate 18 Needle and Syringe Provision (NSP) sites across Tayside, which also provide broader harm reduction services. Health services for PWUD are led regionally through a local managed care network which brings together NHS services, the charity sector, partners from governmental and educational institutions, and representatives from the police service, into a single group to enable multi-dimensional service delivery [36].

### Study design

This cross-sectional study formed the qualitative component of a wider mixed-methods study to develop a behavioural intervention to reduce the likelihood of experiencing NFOD. It involved one-off semi-structured interviews with participants, who were PWUD. Interviews were conducted in person, one-to-one, by research team members with lived experience of drug use (henceforth ‘peer researchers’). The peer researchers were recruited and employed in salaried research positions funded directly by the project. They received certified training in Good Clinical Practice, and were trained and mentored in undertaking qualitative research, including semi-structured interview techniques, by a research-experienced psychologist. Regular formal and informal supervision with the psychologist was sustained throughout the project to reflect upon experiences and learnings, both in conducting research, in how lived experience influenced research practice, and how undertaking the research impacted upon personal wellbeing. Interviews were guided by, but not confined to, an interview schedule (Supplementary File 1), co-developed with peer researcher team members. Participants were encouraged to relate their answers to the context of their most recent NFOD. When the interviews were conducted, Scotland was subject to some restrictions related to Covid-19 [37]. Consequently, interviewers and participants, although in the same room, were socially distanced and wore face coverings.

### Methodological orientation and theory

Given the aims of the wider mixed-methods study, the interview schedule was informed by the COM-B model for Behaviour Change, and it was created taking a deductive approach [38]. The rationale for this was to enable us to take a theoretically informed and structured approach to asking questions which accounted for multiple dimensions of human behaviour in a comprehensive way, and help to identify factors which could subsequently underpin the development of a behavioural intervention to reduce NFOD risk. As mentioned, the schedule was co-developed with peer researchers to ensure it made sense in the context of drug use, and minimise the likelihood of omitting relevant questions. Data analysis and coding was undertaken with an inductive approach, to ensure the analysis was driven by the content of the data. The orientation of the analysis was experiential and essentialist, in that we assumed there were objective truths to be explored in the experiences documented by our participants, and we sought to examine the realities of those experiences, rather than the sense-making processes by which those realities were constructed.

### Recruitment

Participants were recruited from one NSP and one community hub with integrated harm reduction services from June to July 2021 by two peer researchers. These staff were based on site, where harm reduction colleagues signposted service clients to them following clinical interactions where that person had disclosed a recent NFOD consequent to illicit drug use. When interested clients approached the peer research team, the study was explained verbally, written information was provided, any questions were answered, and time to consider participation was agreed. Among those who agreed to take part, written informed consent was obtained by the peer researcher, and the interview was conducted on site. In cases where intoxication impaired an individual’s ability to provide informed consent, they were asked to return at another time. Following interview, participants were proffered £20 (GBP) cash in return for their time.

### Data collection

Participants were assigned study ID numbers to ensure confidentiality. Interviews were digitally recorded and transcribed verbatim using a word processing programme. Following transcription, the transcripts were reviewed against the recordings again to ensure fidelity.

### Data analysis

The transcripts were analysed using thematic analysis at the semantic level [39]. A six-step sequence was followed by two research team members: (1) Interview transcripts were read twice and annotated to foster familiarity; (2) Initial thematic codes were created; (3) Preliminary themes were identified in light of initial coding. In this phase, the researchers discussed their initial identified codes, which led to the identification of recurring overlapping codes and creation of themes. Peer researchers provided feedback and input on themes and codes; (4) Identified themes were reviewed in the context of the full dataset, enabling selection of illustrative quotations which were catalogued under themes and subthemes; (5) Themes were fully defined and allocated final labels; (6) Findings were written up, which continued the examination of themes and the raw data, their relation to each other, and to the research question, thereby creating a narrative supported by the data.

### Ethics

Ethical approval was received from NHS West of Scotland research ethics committee (20WS0183).

## Results

**Fig. 1:**
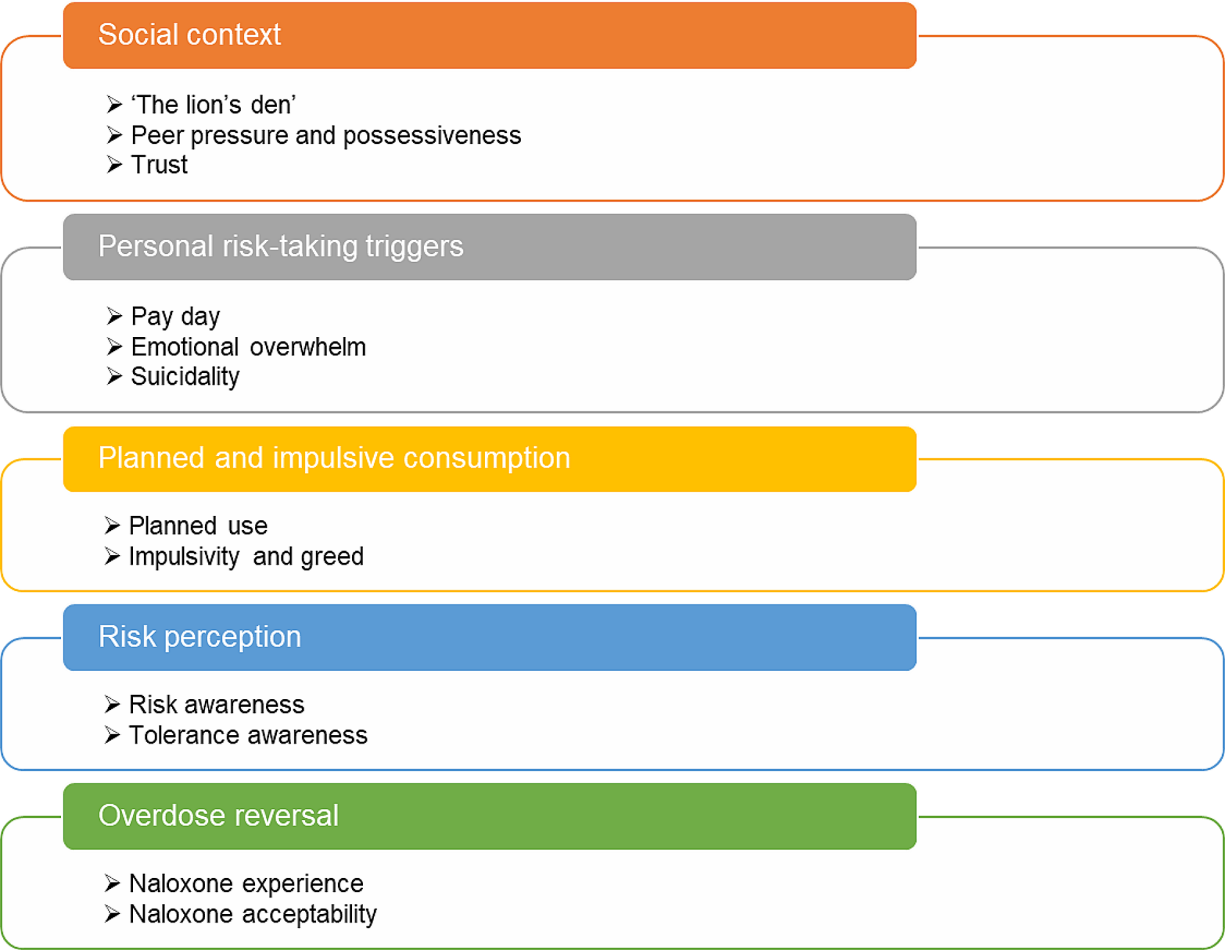
Themes and subthemes identified in the study

Twenty people who disclosed recent illicit drug use were interviewed across the two study sites. Of those, 15 (75%) were male and five (25%) were female, and mean age was 38.2 (SD 7.7) years. Average interview duration was 23 min, and all recordings totalled 450 min. All participants had experienced at least one NFOD in the six months prior to the study, and all disclosed polydrug use, with heroin and crack cocaine use reported alongside both prescribed and illicit use of methadone, diazepam, etizolam, and pregabalin. Five themes were identified in total, within which 12 subthemes were situated (Fig. 1).

Disclosures which illustrate emic definitions of overdose are documented across several subthemes (see ‘tolerance awareness’/‘naloxone experience’); in general terms, experiences were heterogenous. Some hinted at a calm experience, personally experienced. Others related it to a sense of embarrassment, having defined their NFOD consequent to social processes within their wider social group, whilst some did not recall the onset of their overdose, and therefore struggled to define their idea of it:

It is like you just doze out […] It is like you sleep and then you wake up in hospital.

─ 41-year-old male.

I was pretty disappointed in myself […] The only reason I know what happened was getting told by people after a couple of days. And I was pretty shocked.

─ 58-year-old male.

I’ve never seen anybody overdosing and I don’t know what the feelings would be for an overdose. But then when that happened I was like, “what happened there?”.

─ 42-year-old female.

### Social context

Participants described a complex social environment of both helpful and challenging social interactions which they felt impacted their risk of NFOD. This is explored here with reference to the three subthemes identified.

#### Lion’s den

Residing in the ‘lion’s den’, i.e. Tayside’s largest city (Dundee), was seen as a barrier to disconnecting from drug-using acquaintances, for those who wished to reduce or cease their drug use. This was particularly the case following liberation from prison and/or completion of rehabilitation programmes. Some participants attributed the root cause of their NFOD experience to engagement with these acquaintances:

Just out of rehab, I was stuck in the hostel. It just felt like I’d wasted that time coming off the methadone. Sorted my life out, come back to Dundee, and it throws me out in the lion’s den.

─ 42-year-old male (a).

…it’s just been every time I get in the jail then get back out again […] around people that’s got too much drugs and they keep offering us and offering us and offering us.

─ 45-year-old male.

Dundee kills more people every day by drug deaths than the car and you see how many people…like yourself…nobody in the country is like that. Why is Dundee so bad?

─ 36-year-old male.

Getting out of jail and coming back to Dundee. I need to move somewhere where I don’t know anybody.

─ 42-year-old male (b).

Participants related experiences of randomly encountering people they knew, being with a different group of people from usual, and going along with what others in their social group at the time were doing, which increased their exposure to risky drug use:

Most of my pals don’t inject. The thing with the injection the other day was this is a crowd I don’t usually kick about with. “We will get you some units”, and I am just saying, “yes, go for it then”. But I wouldn’t usually normally do that.

─ 49-year-old female.

It must have just been the people I was around. I think I’d probably take a few Valium, then went to someone’s house or something like that…[…] I bumped into somebody and they’ve gave us something else, and then that’s when you can’t remember anything after that.

─ 35-year-old male.

It was about changing the cycle of people, that’s what made me go back into having overdoses.

─ 32-year-old male.

… so you’re banging into different people. Well, I do, ken […]…it just depends on like who you bump into that day.

─ 40-year-old male (b).

#### Peer pressure and possessiveness

Participants disclosed feeling peer pressure from others to buy drugs, which were perceived as ubiquitous despite actively seeking to avoid them, and once purchased, they would consume more than planned. They reported finding it difficult to assert themselves with respect to buying drugs when encouraged to do so, and found it challenging to decline consumption when drugs were present:

It’s not even normally me that wants the gear, it’s the people around me, you know what I mean. […] I say, “look, I’m not interested”, but the peer pressure gets into you.

─ 51-year-old male.

And then, as soon as I got out, I’m at the chemist and guys are there saying, “You want to buy this, do you want to buy that” … and within that day, and that’s me and I’m back on it. And once I pop I can’t stop, ken what I mean?

─ 42-year-old male (a).

You don’t need to go sourcing and that, it comes to you […] You walk through the town centre, I swear to God, there is about 20 or 30 people. […] and there’s no hiding.

─ 44-year-old male.

In particular, participants relayed frustration about other people in their drug-taking network (so-called, ‘payday pals’) knowing when they would receive state benefits and experiencing an increase in peer pressure to buy large quantities of illicit drugs during those times. This frustration appeared to stem from a sense of being taking advantage of, and the transactional nature of such relationships:

I just got paid there a fortnight ago and all the payday pals came out … and where are they now?

─ 49-year-old female.

It just depends who and the situation; who I was sitting with. Because when I get paid everybody is my best mate.

─ 36-year-old female.

… it wasn’t friends I would normally have round. It was only because they knew I’d got paid that day that…they’re only there for the heroin.

─ 42-year-old female.

Spending money on drugs when pressurised by ‘payday pals’ (people who would befriend a participant when they knew that person had purchasing power), brought an interesting, and risky, pressure to use more drugs than usual, given they had paid for the drugs and had a sense of ownership which led to increased consumption:

I’ve been off it [heroin] twelve years and it was just stupid doing it that day. It was my money that was getting spent on it, so I thought, well, you are getting something out of this – what am I getting out of it? Nothing. So I thought, well, I’m taking a hit.

─ 42-year-old female.

… so it’s like, if you can’t beat them, join them. So, I end up taking more because I know if I don’t take it he’s going to take it.

─ 36-year-old female.

You don’t need to take any, you know what I mean. But if I’m paying for it, I’m taking some, you know what I mean?

─ 51-year-old male.

#### Trust

The loose social connections between the participants and some of the people they use drugs with translated to distrust and a sense that they may be prone to experiencing robbery should they experience an NFOD. This vulnerability was compounded by sense that peers would not intervene to help in such situations, instead prioritising financial gain:

Then is anybody going to help you? Probably not. Depending on what company you keep, you know? Have you got money in your pocket? You are getting left and robbed.

─ 49-year-old female.

Some people don’t give a shit about you; they are more interested in the money in your pocket […] I don’t trust any of these freaks.

─ 42-year-old male (b).

But there’s guys that I’ve been with that would just leave me in a alley. Know what I mean? Just leave me there and go through my pockets.

─ 51-year-old male.

When you overdose […] people are in your pockets stealing all your stuff.

─ 41-year-old male.

### Personal risk-taking triggers

Participants recognised that NFOD risk was mediated not only by social context but also by personal triggers. These are explored here within three subthemes, one of which was an automatic psychological association formed by participants, while the two others were spontaneous emotional triggers.

#### Pay day

Similar to the risk associated with the social context of ‘payday pals’, days when state benefits were paid were recognised by participants as a dangerous time for NFOD risk with respect to their own behavioural patterns. This was typically due to buying more drugs than were perceived (retrospectively) to be required, and the potential for polydrug use due to higher purchasing power.

Yeah, I always, when I get my money, I always buy in bulk, I buy in bulk. To make sure that I’ve got [enough] … I get paid on Friday and I’ll buy 1,000 Valium.

─ 30-year-old female.

Yes, the more money you have got the greater risk you are. […] That is when you go and start buying crack.

─ 49-year-old female.

Usually make a plan buying 500 on pay day.

─ 30-year-old male.

When I get paid or he gets paid, that’s the danger times because that’s when we take crack cocaine. That’s when we take heroin to come down you know what I mean, and mixing everything.

─ 36-year-old female.

#### Emotional overwhelm

Feeling unable to cope with unpleasant emotions, and with everyday adversity experienced, was recognised by many interviewees as a trigger for using more drugs, with an associated reduction in their awareness of potential consequences.

If I have a bad day, I go and get more drugs. Set myself up with Valium as well. So it’s another bad thing to do, mixing with heroin. Especially with tablets as well.

─ 32-year-old male.

I was in a really bad place in my head, I suffer from depression, and I was in a bad place in my head, and it seemed like a good idea to take gear you know what I mean, to take me away from it but it didn’t take me away from it, it just made it worse.

─ 51-year-old male.

Because my anxiety and my depression and everything like that, the Valium makes me feel better.

─ 35-year-old male.

#### Suicidality

Feeling emotionally overwhelmed at times reached a tragic point at which some participants admitted to not wanting to continue to live. For some, overdose was considered a peaceful way to end the struggles of life:

I was feeling like that just before I overdosed and I thought, “enough is enough”. You are putting too much of it in, bam, and then overdosed and woke up in hospital with the wires and still awake and I thought, “great, still here” […] I just never wanted to wake up.

─ 41-year-old male.

So…not even depression, just pure absolute, absolute, I’m doing it, I don’t care anymore. I’m done. Nobody bothers with us, I’ve no pals. I’ve got pals that … “associates” … ones that want to see what’s in your pockets. I just had enough. I just thought, no, I’m not doing this anymore, and I just did it. And I was found and then tried it again and I was found. I kept getting found.

─ 36-year-old male.

You know why I did it, right? To kill myself.

─ 27-year-old male.

Mainly, the times I’ve overdosed have been suicide attempts. I’ve never overdosed because of being, like, overindulgent or trying to take too much.

─ 40-year-old male.

### Planned and impulsive consumption

Despite times during which increased usage was triggered as previously described, participants described routine drug use and planned purchases designed to save money and safeguard their wellbeing. Given the high availability of illicit drugs in the region, and the previously described impulsive traits some participants disclosed, these plans for drug consumption over a longer timeframe were not always successfully implemented. These issues are explored here within two subthemes.

#### Planned use

Purchasing in bulk to facilitate future needs (i.e. planned use) was particularly associated with benzodiazepine and heroin use, while crack cocaine was something people purchased spontaneously when they had more money (see Impulsivity and Greed subtheme), and was not typically a planned purchase:

Well, it’s only street Valium I’m addicted to. So, I normally buy, like, 500 for… because it’s cheaper in bulk, so and then I’ll buy…take five every day, and it normally works.

─ 34-year-old female.

You know what, I buy, like, maybe a 100 and try and do that, space them out. 20, 20, 20, 20, 20.

─ 30-year-old male.

But I buy enough to do me a month, so I know I’ve got that everyday… Not always [get to] the end of the month. Not ’til next pay day anyway. […] money is always the trigger.

─ 32-year-old male.

Some participants disclosed a monetary connection to their drug use which belied their planned use, as it was considered a potential trigger for impulsive excessive consumption.

#### Impulsivity and greed

Difficulty in resisting temptation, underpinned by the ease with which drugs can be acquired and associated impulsive purchases of drugs they do not habitually use (e.g. crack cocaine), participants described a self-perceived sense of greed with respect to their drug use. This made it challenging to adhere to their planned use, previously described, and was perhaps linked to, and mediated by, senses of ownership (of the drugs), the social context previously defined, and the emotional overwhelm described:

Because some days I’d prefer to take more heroin than others […] I wouldn’t space it out for the morning or anything. I would just use it.

─ 36-year-old male.

And then if I see somebody who’s got pres (pregabalin), then I buy pres. But then if I’ve got money and I’m out and somebody says they’ve got crack, that’s it. Then I get a message on Messenger saying, “Oh, I’ve got crack”, it’s just tempting and it’s so hard and I only buy a fifty, I only buy a gram and that’s it, then you buy another gram, then you buy another gram.

─ 36-year-old female.

I do try, I do try [to stick to my planned use], but then I get greedy.

─ 30-year-old female.

There is no limit with me, really. There is no limit. The only limit is when I’m KO’d. So I’ll either drink myself to a stupor, snort myself to sleep, or inject myself to an OD.

─ 40-year-old male (a).

### Risk perception

The descriptions of impulsivity and difficulty in sustaining planned drug use led to participants recognising times in which they or someone they knew had been exposed to elevated NFOD risk, and how their awareness of their personal tolerance was lacking. This is explored here across two subthemes.

#### Risk awareness

Risk awareness was linked to descriptions of a lost sense of one’s tolerance following a period of abstinence; using alone; and low quality or purity of drugs. Some of these risks can be mitigated against, such as perhaps consuming drugs as part of a group, or with a friend who was not part-taking, but others were outwith participants’ control:

I had just got out of jail and obviously my tolerance had gone down, and I take too much.

─ 42-year-old male (b).

Risky business I know [using alone]. I’m on my own. […] I’ll just take a bit at a time, just so that I make sure I don’t go over.

─ 51-year-old male.

I have been getting stuff and some stuff’s been like … I will say to her [partner], “I will go first”, because if it’s shite and anything happens I would rather I died than she did.

─ 41-year-old male.

Could just be the kit as well, you get all different kinds of kit [and some] could be stronger than others.

─ 33-year-old male.

#### Tolerance awareness

Experiences with respect to dose tolerance were varied. Despite some participants following harm reduction advice they had received and splitting doses, interviewees disclosed finding it difficult to know when to stop (i.e. how far could the risk be pushed before experiencing NFOD). Some participants reflected on some physical symptoms alarming them about an imminent loss of consciousness, whilst others reported feeling fine but subsequently waking up with first responders attending to them:

You think you’re okay and then the next minute you wake up and there’s ambulance people around you. And you’re like that fff. I thought I was okay, ken.

─ 44-year-old male.

I can’t see it every time, no. There’s times when it’s too late. There’s times when it’s too late and you’ve done it now. You’re at a stage where you can’t get yourself up and walk in a straight line and that’s when you know you’re…that’s when I’ve taken an overdose.

─ 32-year-old male.

The pins and needles in my arm. In my feet. Then onto your head. That’s when I get scared anyway. Just before it comes on.

─ 40-year-old male (b).

Because I know my limit, right. I know my limit, how many to take, I don’t take any more than 20 ever. If I take over 20 then I’d be worried.

─ 30-year-old male.

I get like a, it’s like a feeling in my head, tingling in my head…And that kind of feeling, and that’s when I know, oh wait a minute, like that’s too much.

─ 36-year-old female.

Because obviously I was clean and I didnae realise how clean I was, know what I mean, the high risk.

─ 42-year-old male (a).

### Overdose reversal

Awaking to emergency first responders was shared by many as a shocking and confusing experience following an NFOD. Naloxone had been administered in all participants’ overdose reversal. Despite the shocking and confusing nature of resuscitation by Naloxone described by interviewees, acceptability for its use was universally high, with participants disclosing feelings of gratitude toward first responders.

#### Naloxone experience

Participants recounted a sense of shock at regaining consciousness with first responders surrounding them, associated with feelings of confusion and sometimes anger as a good high had been had interrupted. Some participants further reported immediate drug-seeking behaviour following regaining of consciousness after Naloxone receipt:

Oh it was freaky! When I woke up and everybody was around us […] It was spacey. The ambulance people are trying to keep you down and you’re trying to get up. And the ambulance are going, “please just come to the hospital”, and I’m going, “I don’t need the hospital! I’m strong now, I need to get something else!”

─ 44-year-old male.

It was weird…when I came around it’s all sort of strange at first. Sort of rattle, you know what I mean. And I lost my temper a wee bit, you know what I mean? “But you overdosed”, I had no choice, and I waited an hour and went and scored again.

─ 51-year-old male.

Just everybody was round me and that was it and obviously you know what it is like just wakening and thinking […] It is like I was coming back from the clouds.

─ 27-year-old male.

As noted, the high was often sought again immediately after, increasing the risk of repeated NFOD, despite participants being aware of the risk. Yet, on reflection, most participants expressed gratitude to those who had reversed their overdose using Naloxone.

Horrible, horrible feeling, but I was grateful. I was grateful in the end, after the effects wore off. One of the times I ended up using more drugs then went into overdose again.

─ 32-year-old male.

I was glad actually, even though there was a pure instant rattle after it. But I was still glad because I would be dead if they didn’t use the Naloxone.

─ 42-year-old female.

When I came around […] I was hanging off the ambulance man’s neck in the ambulance saying, “Thank you! Thank you! Thank you!”, like cause if it wasn’t for you….

─ 30-year-old female.

#### Naloxone acceptability

That sense of gratitude was associated with a universal acceptance and support for Naloxone carriage and administration by peers, despite participants’ awareness of possible negative sensations immediately following administration. The anticipated regret of not saving someone’s life outweighed the possibility of negative experiences, which therefore did not deter participants’ intentions to use Naloxone when needed by people they knew. This conflicted somewhat with previously highlighted issues regarding trust in peers to respond to an overdose:

They need it if they’re going to die, it’ll be on your conscience.

─ 34-year-old female.

Even though they go mental because they’re going into an instant rattle – they’re pure wanting to fight with you, man – but I’d still stab them with it. I don’t care; as long as I’m thinking I’m saving their life.

─ 42-year-old female.

Because you are saving a life, so you have got to use it. You need to use it. What else are you meant to do? Let them die?

─ 41-year-old male.

## Discussion

In this cross-sectional qualitative study, undertaken in Tayside, Scotland, we sought to understand the lived experiences of NFOD among PWUD in the region. Three-quarters of participants were male, and the average age was 38 years; these demographics mirror those of Scottish cohorts reported elsewhere [40]. All had experienced NFOD in the last six months and reported polydrug use, which included a mixture of both prescribed and illicit (or illicitly obtained) drugs. We identified five intersecting themes: social context, personal risk-taking triggers, planned and impulsive consumption, risk perception, and overdose reversal. These themes spoke to the critical environmental, behavioural, cognitive, emotional, economic, and marketplace, factors which influence the risk of experiencing NFOD in the region. We believe this work provides useful insights for health and social care practitioners, as well as policy makers, seeking to reduce the harms associated with illicit drug use by exploring reforms to provision of healthcare, social care, and carceral services; the regulatory environment for drugs; and the physical environments in our towns and cities. We also believe the work provides useful insights into the lived experience underlying risk factors previously noted in the introduction, as well as insights into recovery from NFOD and experiences of naloxone. Fundamental to all our participants’ experiences of NFOD were peer-to-peer dynamics, psychological burden, and changeable social contexts.

Known risk factors for NFOD such as liberation from prison, recently ceased addictions treatment, polydrug use, and social dynamics, could be explored through our social context theme. Our participants described exiting detoxification programmes and being liberated from prison into myriad situational stressors which precipitated risky drug use. This speaks to the enduring need for prison, health, and social services to ensure robust social supports which enable people to avoid risky social contexts and reduce their risk of harm in this vulnerable period. Participants further outlined peer pressure and narratives of possession (paying for drugs, but not getting their share) and manipulation (payday pals) which illustrate key social dynamics in the context of seeking to avoid drug use, and in using more than intended, which underlies risks identified with polydrug use and social dynamics.

Residing in Dundee – the ‘lion’s den’ – created a sense of entrapment which has been similarly reflected in previous research that explored notions of place as mediating drug use risk. For example, Australian research explored this where initiation into drug injecting, and risky drug use, was closely linked with social connections to experienced injectors [41]. Similar work from India found peer influence – typically a friend in the context of long-established social networks – determined initiation into injection drug use, as well as risky drug-taking behaviours which increase risk of NFOD [42]. Such manifestations of peer pressure was also reported by our interviewees: even if wishing to refrain from drug use, they reported yielding to the social context which is a phenomenon, coined ‘peer persuasion’, also observed in research conducted in Moldova, where a participant stated, for example, ‘I don’t know how I agreed, but I agreed’, to injection drug use [43]. This raises an interesting question; to what extent can harm be reduced by peer interventions, if peer-to-peer social processes may induce risk for some people in the first place? Antecedent risk may be diminished further upstream through robust supports which explore individuals’ social connectedness (i.e. group identification) and how that may be reframed as part of a recovery-based care approach within harm reduction services.

Our cohort further connected the peer pressure they experienced to purchasing power and impulsivity, through their descriptions of ‘payday pals’ and injecting drugs despite not intending to do so, including in unfamiliar environments which can induce overdose even at usual dosage due to environmental psychological stressors [44, 45]. This purchasing power, linked to synchronised state income payments, has been associated with increases in high-intensity drug use and cognate increases in NFOD and fatal overdose [46, 47]. Some research has suggested staggered payment schedules may mitigate escalations in drug use and associated unintended harms [48]. Our study contributes to this existing work by elucidating the context further; our participants reported wishing to refrain from drug use, whilst their payday pals engaged, which led to feelings of manipulation that drove subsequent impulsive consumption of a higher-than-usual quantity, or use in unfamiliar settings, leading to subsequent NFOD. NFOD risk associated with drug use in unfamiliar settings may be ameliorated by provision of safer consumption facilities – as yet unavailable in Scotland, though work is ongoing and key decision makers are supportive – which can provide a familiar setting for drug use and have been shown to reduce risk of NFOD and other drug-related harms [49, 50].

The sense of manipulation and peer pressure was compounded by a lack of trust, with some interviewees feeling their peers may prioritise monetary gain over their recovery from an NFOD. It is possible this lack of trust may be a manifestation of prior traumatic experiences common among PWUD [51, 52]. There are multiple reports summarising the traumatic experiences of PWUD from childhood through to adulthood, with some work directly linking childhood trauma to subsequently experiencing NFOD [4, 53–56]. There was a surprising dissonance between the lack of trust which drove interviewees to questions their peers’ motivations – suspecting they may prioritise monetary gain, as mentioned – and the expectation interviewees placed upon themselves to administer Naloxone to a peer in need. This may speak to the critical role that received stigma plays in perpetuating (mis)trust among PWUD – something not commonly reported in existing literature – and a related necessity for further public health campaigns to promote empathy and reduce the stigma of addiction [57]. Recent years have seen Naloxone supply scaleup significantly among PWUD in Scotland, and participants in our study indicated high willingness to administer, and be administered it, despite the dissonance identified. Few studies have evaluated the psychological impact upon peers of naloxone provision, and inhabiting the competing roles of both provider and recipient of naloxone can cause emotional stress and difficulties maintaining personal wellbeing [58]. These findings suggest we may need to consider whether naloxone programmes can be optimised to deliver more than training to reverse overdose, perhaps integrating psychological supports for peers and training peers to deliver stabilising interventions which reduce risky drug taking behaviours in the first place. Fundamental to these will be involving peers in the design and decision making for such programmes at the outset.

Beyond specific social dynamics, recent evidence determined that people experiencing problematic drug use (relative to those who do not) have significantly greater difficulties in regulating emotions, particularly regarding strategic thinking and impulsivity [59]. This evidence aligns with our work, where we found emotional overwhelm and associated impulsive poly-drug use, identified in existing literature as risk factors for NFOD, to be closely linked experiencing NFOD. This was despite participants sometimes having planned their use over time by purchasing specific quantities. Such cases of emotional overwhelm appeared to be driven by a struggle to cope with everyday adversity, and experiences of depression, and somewhat determined intentionality regarding consumption leading to NFOD, with some participants disclosing suicidal ideation immediately prior to their (intentional) overdose. Suicidal ideation is common among PWUD, with 22% (19-25%) estimated to have ever attempted suicide [4, 60]. Some of our participants described intense suicidal ideation prior to their NFOD. When considered alongside other complex social dynamics reported around trust, risk perception, entrapment, and manipulation among peers, we recommend substantial scaleup of mental health and suicide prevention services which proactively address emotional overwhelm, perhaps through low-threshold access to safety and stabilisation work, as a critical need to reduce the risk of NFOD [51, 61]. Ideally situated within a psychosocial care framework which holistically supports PWUD.

Easy access to the quantity of drugs required to induce NFOD was common, as participants perceived local drug supply to be abundant, with opportunity to purchase frequent both in everyday settings and close to healthcare environments such as community pharmacies. Availability is not so straightforwardly described, however. Incessant targeting of PWUD by dealers has been observed in the literature previously, and our work highlights how the enduring criminalisation of certain drugs works to increase the harms associated with using them [62]. Our participants described a physical inability to ‘hide’ from the open drug market, peer pressure to participate in the procurement of drugs (conflicting with their intentions), and active targeting by dealers at health venues which ought to be safe spaces. Health and police authorities, as well as local government, would benefit from consideration of the evidence here when designing policing strategies for the benefit of community public health. This a stated priority of the Scottish police force, and current approaches appear to be struggling to meaningfully reduce the risk of NFOD among PWUD, despite police carriage of naloxone [63]. Additional gains may be made in scoping the potential for public health approaches which integrate mental health and wellbeing responses into community policing, given the burden among PWUD. However, currently Police Scotland staff currently receive little training in this regard, and officers report a lack of strategic leadership on the issue [64].

Heightened NFOD risk awareness was closely linked by participants to liberation from prison, using drugs alone, the quality of drugs procured, and perceptions of dose tolerance. Liberation from prison has been widely recognised as a critical risk factor for NFOD, particularly in the first two weeks [65, 66]. Our participants described being liberated directly into existing social contexts which immediately heightened the likelihood of risky drug use, particularly as some also described not knowing ‘how clean’ they were. Further than that, they were often liberated into social environments characterised by widespread drug availability, ongoing use, and attendant social processes which drive risk. None reported experience of transitional arrangements which could help to minimise the likelihood of experiencing harms. It is critical that carceral services acknowledge this context and fulfil their duty to the health of people in their care, and the wider community, by improving links to community services for PWUD during liberation. Similarly, the literature on using alone aligns with our participants’ perceptions. Reasons for using alone vary from the availability of drugs/funds, to avoiding sharing, to senses of personal safety, stigma, and shame [67]. Among our participants, it was driven by drug availability and motivations not to share drugs with others. However, doing so brings with it substantial risk. Close to half of people who experienced fatal overdose in Dundee in recent years were alone at the time, and reports estimate that using alone confers up to 75% higher odds of NFOD [53, 68]. Our participants described knowing it was risky to use alone, and taking extra care not to overdose, yet still experiencing one.

In light of our findings, future research may seek to explore interventions to assist in reducing impulsivity in drug use contexts, particularly in the context of individuals not wishing to use drugs whilst their peers did so, as several of our participants reported. Additionally, the role of received stigma in NFOD risk was noted in our results, but remains underexplored in the literature. A recent study suggested internalised stigma confers 51% higher odds of NFOD, and future research could explore the social and psychological mechanisms through which received stigma contributes to NFOD risk, and strategies to mitigate this [69]. Finally, optimised transitional care models upon community re-entry from detoxification programmes and incarceration warrant further exploration; these are vulnerable time periods and no optimal approach, which is adaptable at scale, is clearly apparent in the literature. Collaborative research exploring new options integrating health, social, and carceral, authorities could add valuable evidence on new approaches to reducing risk.

### Limitations

This study has several limitations. First, the cohort consisted of a highly localised, fairly homogenous, sample of PWUD. This inherently limits the wider transferability of the observations. However, as noted, many of the experiences disclosed align with risk factors identified in international contexts. Second, there was a preponderance of male participants in our cohort. Although the proportions broadly reflect the local cohort of PWUD in Tayside, we acknowledge that the experiences disclosed may not reflect events in the lives of women who use drugs who often experience NFOD in a context of psychological or physical violence, including intimate partner violence, or violence in the context of sex work; an experience not disclosed in our work [70, 71]. Finally, although we took an experiential perspective, to ensure the work was grounded in the lived experience of PWUD, our interview guide was informed by a behaviour change model which, in classifying behaviour into capability, opportunity, and motivation, may miss some of the inherent variability which exists in human behaviour, thereby limiting the responses which may be elicited from respondents accordingly. We tried to mitigate against this by co-producing our guide with peer researchers with lived experience of NFOD, to ground the work in an experiential perspective.

## Conclusions

In this cross-sectional study conducted in Tayside, Scotland, we recruited twenty PWUD and interviewed them about their experiences of NFOD. We identified five key themes and the results spoke to the critical factors which influence the risk of experiencing NFOD in the region. We believe this work can be used by policy makers and healthcare providers to influence decision making to improve the health and wellbeing of PWUD. For example, by pushing for implementation of safer consumption facilities to reduce the likelihood of people using drugs in unfamiliar environments, which heightens NFOD risk; exploring the potential for people to opt-in to staggered state income payments to reduce risk; implementing low-intensity psychosocial interventions across addictions services focussed on safety and stabilisation, and emotional regulation, with adequate staffing to deliver these; reforming prison liberation processes to ensure adequate transitional supports in the community; regularly delivering public health initiatives to reduce stigma; and pushing for reform to the Misuse of Drugs Act 1971 to disrupt illicit supply and minimise the targeting of people living with addiction by dealers or, in the absence of this, ensuring police services are implementing public health approaches to reduce such targeting. Furthermore, we hope to use this work, alongside other ongoing research, to inform development of a behavioural intervention to mitigate against the risk of experiencing NFOD subsequent to illicit drug use.

### Electronic supplementary material

Below is the link to the electronic supplementary material.

Supplementary Material 1

## Data Availability

Consideration will be given to sharing the data underpinning this study upon receipt of a proportionate and methodologically sound data access request. Such requests should be sent, in the first instance, to the corresponding author.

## Abbreviations

GBP: Great British Pounds
NHS: National Health Service
NSP: Needle and Syringe Provision
NFOD: Non-fatal overdose
OAT: Opioid agonist therapy
PWUD: People who use drugs
