# Supplementary material for: ‘It was like coming back from the clouds’: a qualitative analysis of the lived experience of overdose consequent to drug use among a cohort of people who use drugs in Scotland
Source: Harm Reduct J. 2024 Jun 7;21:112. doi: 10.1186/s12954-024-01033-7 (PMC11157918; doi:10.1186/s12954-024-01033-7)
Supplement: Supplementary file 1 — Supplementary Material 1 [file 12954_2024_1033_MOESM1_ESM.docx]

**Supplementary File 1**

**Title**

‘It was like coming back from the clouds’: a qualitative analysis of the lived experience of overdose consequent to drug use among a cohort of people who use drugs in Scotland.

**Authors**

Christopher J Byrne^1,2^ Fabio Sani^3^ Teresa Flynn^4^ Amy Malaguti^3,5^

**Affiliations**

^1^Division of Molecular and Clinical Medicine, School of Medicine, Ninewells Hospital and Medical School, University of Dundee, Dundee, UK

^2^Directorate of Public Health, NHS Tayside, Kings Cross Hospital, Dundee, UK.

^3^Division of Psychology, Scrymgeour Building, School of Humanities, Social Sciences and Law, University of Dundee, Dundee, UK

^4^Hillcrest Futures, Explorer Road, Dundee, UK.

^5^Tayside Drug and Alcohol Recovery Psychology Service, NHS Tayside, Dundee, UK

**INTERVIEW TOPIC GUIDE**

**Title**: Designing a Behaviour Change Intervention to Reduce the Risk of Overdose

BROAD TOPICS TO DISCUSS AT INTERVIEW (COM-B Model)

***Experience of non-fatal overdose***

*Physical capability*

Person’s sense of own health, their skill level of using drugs/ injecting and of using naloxone.

*Psychological capability*

Person’s mental functioning, their understanding of actions & consequences of injecting higher doses, recognising high risk situation, recollection of past experiences and useful information (i.e. where naloxone is stored).

*Reflective motivation*

Conscious thought processes involved in plans to use drugs, evaluations of what to use and when, evaluations of personal needs and safety.

*Automatic motivation*

Habitual & instinctive motivation, emotional influences, desired and habits around drug consumption and keeping one safe which are not consciously processed.

*Physical opportunity*

Environment structure, financial and material resources, time of month they get paid, amounts of drugs available and purchased, availability and prompting for take-home naloxone when using services.

*Social opportunity*

Influence of other people and other organisations. Using drugs with others or alone, social norms and acceptability of naloxone use, consequences on relationship after naloxone use, cultural norms related to drug use, services’ norms for asking clients about possession of naloxone.

**INTERVIEW SCHEDULE**

**Title**: Designing a Behaviour Change Intervention to Reduce the Risk of Overdose

1. Tell me a bit about **your** own experience of overdose.
2. What was going on for you at the time when you overdosed?
3. How were you feeling at the time just before you overdosed?
4. Do you know if you’ve reached your tolerance/limits level? And if you are mixing?
   1. If YES: What was different about your last overdose? (Place / Emotions / Time)

If NO: Can you explain why you went over your tolerance limit?

1. When do you feel you are most at risk of overdosing?
2. How confident are you that you could recognise you are at risk of overdosing in that moment? / Why?
3. How do you source drugs and do you plan on your use? – Explain plan
4. What happened to this habit/plan when you overdosed? Was anything different than usual?
5. How do you decide what drugs to buy?
6. How do you decide how much to buy?
7. How do you decide from where to buy?
8. How do you decide how much to use at one time?
9. How skilled are you at injecting yourself?
10. What makes using with some people more overdose-risky than others?
11. How confident are you in using Naloxone? / Do you know how to use Naloxone?
12. How acceptable is it to use Naloxone on a friend/associate/partner?
13. What was it like for you when somebody used Naloxone on you?
14. How acceptable is it for someone to use Naloxone on you?
15. Is there anything else that you think it’s important to tell me about your experience of overdose?

Hints/Tips:

Differentiate between personal and friend/family experience of overdose.

Behaviour & Emotions just before overdosing.

Other people / places involved .
